# PGC-1α supports glutamine metabolism in breast cancer

**DOI:** 10.1186/2049-3002-1-22

**Published:** 2013-12-05

**Authors:** Shawn McGuirk, Simon-Pierre Gravel, Geneviève Deblois, David J Papadopoli, Brandon Faubert, André Wegner, Karsten Hiller, Daina Avizonis, Uri David Akavia, Russell G Jones, Vincent Giguère, Julie St-Pierre

**Affiliations:** 1 Goodman Cancer Research Centre, McGill University, 1160 Pine Ave. West, Montréal, PQ H3A 1A3, Canada; 2 Department of Biochemistry, McGill University, 3655 Promenade Sir-William-Osler, Montréal, PQ H3G 1Y6, Canada; 3 Department of Physiology, McGill University, 3655 Promenade Sir-William-Osler, Montréal, PQ H3G 1Y6, Canada; 4 Luxembourg Centre for Systems Biomedicine, University of Luxembourg, 7 Avenue des Hauts-Fourneaux, Esch-Belval L-4362, Luxembourg; 5 Metabolomics Core Facility, McGill University, 1160 Pine Ave. West, Montréal, PQ H3A 1A3, Canada; 6 Department of Biological Sciences, Columbia University, 1212 Amsterdam Ave, New York, NY 10027, USA; 7 Center for Computational Biology and Bioinformatics, Columbia University Medical Center, 1130 St. Nicholas Ave, New York, NY 10032, USA

**Keywords:** PGC-1α, Glutamine, Metabolism, Breast cancer, Metabolomics

## Abstract

**Background:**

Glutamine metabolism is a central metabolic pathway in cancer. Recently, reductive carboxylation of glutamine for lipogenesis has been shown to constitute a key anabolic route in cancer cells. However, little is known regarding central regulators of the various glutamine metabolic pathways in cancer cells.

**Methods:**

The impact of PGC-1α and ERRα on glutamine enzyme expression was assessed in ERBB2+ breast cancer cell lines with quantitative RT-PCR, chromatin immunoprecipitation, and immunoblotting experiments. Glutamine flux was quantified using ^13^C-labeled glutamine and GC/MS analyses. Functional assays for lipogenesis were performed using ^14^C-labeled glutamine. The expression of glutamine metabolism genes in breast cancer patients was determined by bioinformatics analyses using The Cancer Genome Atlas.

**Results:**

We show that the transcriptional coactivator PGC-1α, along with the transcription factor ERRα, is a positive regulator of the expression of glutamine metabolism genes in ERBB2+ breast cancer. Indeed, ERBB2+ breast cancer cells with increased expression of PGC-1α display elevated expression of glutamine metabolism genes. Furthermore, ERBB2+ breast cancer cells with reduced expression of PGC-1α or when treated with C29, a pharmacological inhibitor of ERRα, exhibit diminished expression of glutamine metabolism genes. The biological relevance of the control of glutamine metabolism genes by the PGC-1α/ERRα axis is demonstrated by consequent regulation of glutamine flux through the citric acid cycle. PGC-1α and ERRα regulate both the canonical citric acid cycle (forward) and the reductive carboxylation (reverse) fluxes; the latter can be used to support *de novo* lipogenesis reactions, most notably in hypoxic conditions. Importantly, murine and human ERBB2+ cells lines display a significant dependence on glutamine availability for their growth. Finally, we show that PGC-1α expression is positively correlated with that of the glutamine pathway in ERBB2+ breast cancer patients, and high expression of this pathway is associated with reduced patient survival.

**Conclusions:**

These data reveal that the PGC-1α/ERRα axis is a central regulator of glutamine metabolism in ERBB2+ breast cancer. This novel regulatory link, as well as the marked reduction in patient survival time associated with increased glutamine pathway gene expression, suggests that targeting glutamine metabolism may have therapeutic potential in the treatment of ERBB2+ breast cancer.

## Background

Altered metabolism is an emerging hallmark of cancer cells [1]. The most studied metabolic alteration of cancer cells is their high reliance on glycolysis even if there is sufficient oxygen to support mitochondrial oxidative phosphorylation (Warburg effect). The rapid ATP production of glycolysis is thought to benefit the enhanced proliferation of cancer cells. However, glutamine metabolism has been increasingly recognized as a central metabolic pathway in cancer, and it is thought that the catabolic and anabolic roles of glucose and glutamine cooperate in fuelling tumor growth [2]. The discovery that the oncogene Myc is an important regulator of glutamine metabolism has further strengthened the importance of this metabolic pathway in cancer [3,4].

Recent studies revealed that reductive carboxylation of glutamine to citrate through reversal of the citric acid cycle (CAC) and the subsequent use of citrate for lipogenesis constitute a key anabolic route in hypoxia in cancer cells, as well as in cancer cells with mitochondrial dysfunctions [5-8]. Hypoxia-induced factor 1 (HIF-1) plays an important role in this metabolic reprogramming, as cells constitutively expressing HIF-1α perform reductive carboxylation of glutamine in normoxia [5,7]. Clearly, the ability to rely on this metabolic pathway will be a distinct advantage for solid tumors that experience fluctuating levels of oxygen by permitting elevated production of lipids, which are important notably for membrane biogenesis.

The family of peroxisome proliferator activated receptor gamma coactivator 1 (PGC-1) consists of three members, PGC-1α, PGC-1β, and PRC. These transcriptional coactivators play a central role in metabolism [9,10]. Even though the importance of the PGC-1s in regulating mitochondrial functions and metabolism is well established in general, their roles in regulating cancer metabolism and proliferation have only started to emerge recently. The PGC-1s have been shown to have both pro- and anti-tumorigenesis effects [11,12]. In breast cancer, PGC-1α has been shown to promote the growth of ERBB2+ tumors *in vivo* and to regulate their mitochondrial metabolism and angiogenic properties [12,13]. In this paper, we inquired whether PGC-1α participates in the control of glutamine metabolism in breast cancer. We reveal that PGC-1α increases the expression of glutamine metabolism genes, augments glutamine-mediated forward and reverse CAC fluxes, as well as elevates glutamine-mediated lipogenesis in hypoxia. This PGC-1α-mediated increase in glutamine metabolism will confer an advantage to solid breast cancer tumors experiencing limited nutrients and oxygen supply. Relevance to the human disease is underlined by the findings that PGC-1α expression is positively correlated with that of the glutamine pathway in ERBB2+ breast cancer patients, and that high expression of the glutamine pathway is associated with shorter survival time.

## Methods

### Animals

All procedures were conducted in accordance with approved animal protocols by the McGill University Animal Care Committee. Wild-type mice (C57BL/6J) and muscle creatine kinase (mck)-PGC-1α transgenic mice (C57BL/6-Tg(Ckm-Ppargc1a)31Brsp/J) as described in [14] were purchased from The Jackson Laboratory (Bar Harbor, ME, USA). All animals used were female and were sacrificed at approximately 12 weeks of age.

### Tissue culture

Stable cell lines Control-1, PGC-1α-1.1, and PGC-1α-1.2 have been described in [13]. These cells were cultured in Dulbecco's Modified Eagle's Medium (DMEM), 10% FBS, 10 μg/mL insulin, 20 mM HEPES, penicillin/streptomycin, 1 μg/mL puromycin, 400 μg/mL G418, at 37°C and 5% CO_2_. SK-BR-3 and BT-474 were obtained from the American Type Culture Collection (ATCC) and cultured in DMEM supplemented with 10% FBS and penicillin/streptomycin. Hypoxia experiments were performed in a Thermo Scientific (Rockford, IL, USA) Heraeus HERAcell 150 set at 1% O_2_ and 5% CO_2_. Pharmacological inhibition of ERRα was performed using Compound 29 (C29) at 5 μM (0.1% DMSO). C29 was synthesized according to [15].

### Gene expression

Total RNA from cultured cells, grown to 50% to 60% confluence in 35 mm plates, was extracted using the Aurum Total RNA Mini Kit (Bio-Rad, Mississauga, Canada) and was reverse transcribed with iScript cDNA Synthesis kit (Bio-Rad). mRNA expression analyses by real-time PCR were performed using iQ SYBR Green Supermix (Bio-Rad) and gene-specific primers with the MyiQ2 Real-Time Detection System (Bio-Rad). Values were normalized to TATA binding protein (*Tbp)* for murine cell lines or beta-2 microglobulin (*B2M*) for human cell lines.

### Chromatin immunoprecipitation

Chromatin was prepared from PGC-1α-1.1 cells, using approximately 10^7^ cells per chromatin immunoprecipitation (ChIP). ChIP was performed as described previously [16] using a specific anti-PGC-1α antibody (Santa Cruz Biotechnology, Inc., Dallas, TX, USA, catalog #sc-13067) or specific anti-ERRα antibody (Epitomics, Burlingame, CA, USA, catalog #2131-1). Quantification of PGC-1α or ERRα ChIP enrichment was performed using real-time PCR using the LightCycler 480 instrument (Roche, Mississauga, Canada) and specific primers for genomic regions. ChIP enrichment was normalized against the control region and further normalized against the IgG control.

### Immunoblotting

Total proteins from cultured cells or muscle tissue were extracted with lysis buffer (50 mM Tris–HCl pH 7.4, 1% Triton X-100, 0.25% sodium deoxycholate, 150 mM NaCl, 1 mM EDTA) with inhibitors (2 μg/mL pepstatin, 1 μg/mL aprotinin, 1 μg/mL leupeptin, 0.2 mM phenylmethylsulfonyl fluoride and 1 mM sodium orthovanadate) and quantified with the Bio-Rad Protein Assay kit (Catalog #500-0006). The blots were incubated according to the manufacturer's instructions with the following primary antibodies: Glud1 (GeneTex, Irvine, CA, USA, GTX88164), Gls (Abcam, Toronto, Canada, AB93434), Got1 (GeneTex, GTX88903), Got2 (GeneTex, GTX88925), Hsp90 (Santa Cruz Biotechnology, sc-101494), and Actin (Santa Cruz Biotechnology, sc-1616) and with horseradish peroxidase-conjugated secondary antibodies (GE Healthcare, Mississauga, Canada, or Santa Cruz Biotechnology). The results were visualized using Western Lightning Plus-ECL (Perkin Elmer, Waltham, MA, USA) or SuperSignal West Femto ECL (Pierce, Rockford, IL, USA). Densitometry analyses were conducted using ImageJ software (NIH).

### siRNA

BT-474 cells were subjected to either 10 nM control siRNA (Dharmacon, Rockford, IL, USA, D-001810-10-05) or a combined 10 nM pool of four siRNA specifically targeting PPARGC1A (Qiagen, Germantown, MD, USA, FlexiTube GeneSolution GS10891). Cells were transfected using calcium phosphate (CaPO_4_) and incubated for 120 h before extraction.

### ^14^C-lipid incorporation assay

Cells were seeded at a density of 400,000 cells per 35 mm plate in standard growth media and conditions. After 24 h, the medium was supplemented with trace amounts of [U-^14^C]-glutamine (0.094 μCi/mL) and the cells were further incubated either in normoxia (21% O_2_) or hypoxia (1% O_2_) for 24 h (Control-1 and PGC-1α-1.1) or 48 h (BT-474). Individual experiments were conducted in triplicate with additional plates for cell counting. Cells were washed with PBS, collected using 0.5% Triton X-100, and stored at −80°C. After thawing, lipids were extracted using chloroform/methanol; the lipid-rich chloroform phase was dried under air and resuspended in 150 μL chloroform. Scintillation counts were measured over 5 minutes per sample using a MicroBeta2 scintillation counter (Perkin-Elmer) and Eco-Lume reagent (MPBio, Solon, OH, USA). Counts were normalized for cell number.

### Glutamine uptake

Cellular glutamine uptake was measured using the BioProfile 400 analyzer (Nova Biomedical Corp., Waltham, MA, USA). Following growth under normoxic (21% O_2_) or hypoxic (1% O_2_) conditions, media were removed and maintained on ice until analysis. Glutamine uptake was calculated as the difference in glutamine content between culture media and unseeded media incubated in parallel plates and normalized for cell count.

### Mass isotopomer distribution analysis

The metabolomics analyses by GC/MS were adapted from [17]. Additional details are provided in Additional file 1.

### The Cancer Genome Atlas analyses

Breast cancer data, clinical data, and survival data for 732 patients were obtained from The Cancer Genome Atlas (TCGA) [18]. RNAseq data were downloaded from TCGA web portal using the reads per kilobase of exon model per million mapped reads (RPKM) quantification [19]. RPKM data was log2 transformed and expression values lower than 0.25 RPKM were removed. To generate an overall pathway score, all nine genes in the glutamine metabolism pathway were Z-transformed (mean 0 and standard deviation of 1). Mean of zero corrects for the fact that the genes may be expressed at different levels. Transforming the standard deviation to 1 causes the genes to change in the same magnitude, so we can see if the overall pathway is over/underexpressed. If the genes are expressed at different levels, the changes in the pathway will be dominated by the genes that were originally expressed at higher levels. We used the median of Z-scores to generate a pathway score, which was used in correlation and survival analysis.

### Statistical analyses

All statistical analyses were performed using GraphPad Prism (GraphPad Software, Inc., CA, USA), Microsoft Excel (Microsoft Corporation, CA, USA) or Matlab (The Mathworks, Inc., MA, USA). Survival analysis was calculated using an implementation of Kaplan-Meir log rank testing from Matlab Exchange [20].

### Additional methods

Additional methods are provided in Additional file 1 and in Additional file 2: Table S1.

## Results

### PGC-1α modulates the expression of enzymes involved in glutamine metabolism in ERBB2/Neu-induced breast cancer cells

**Figure 1:**
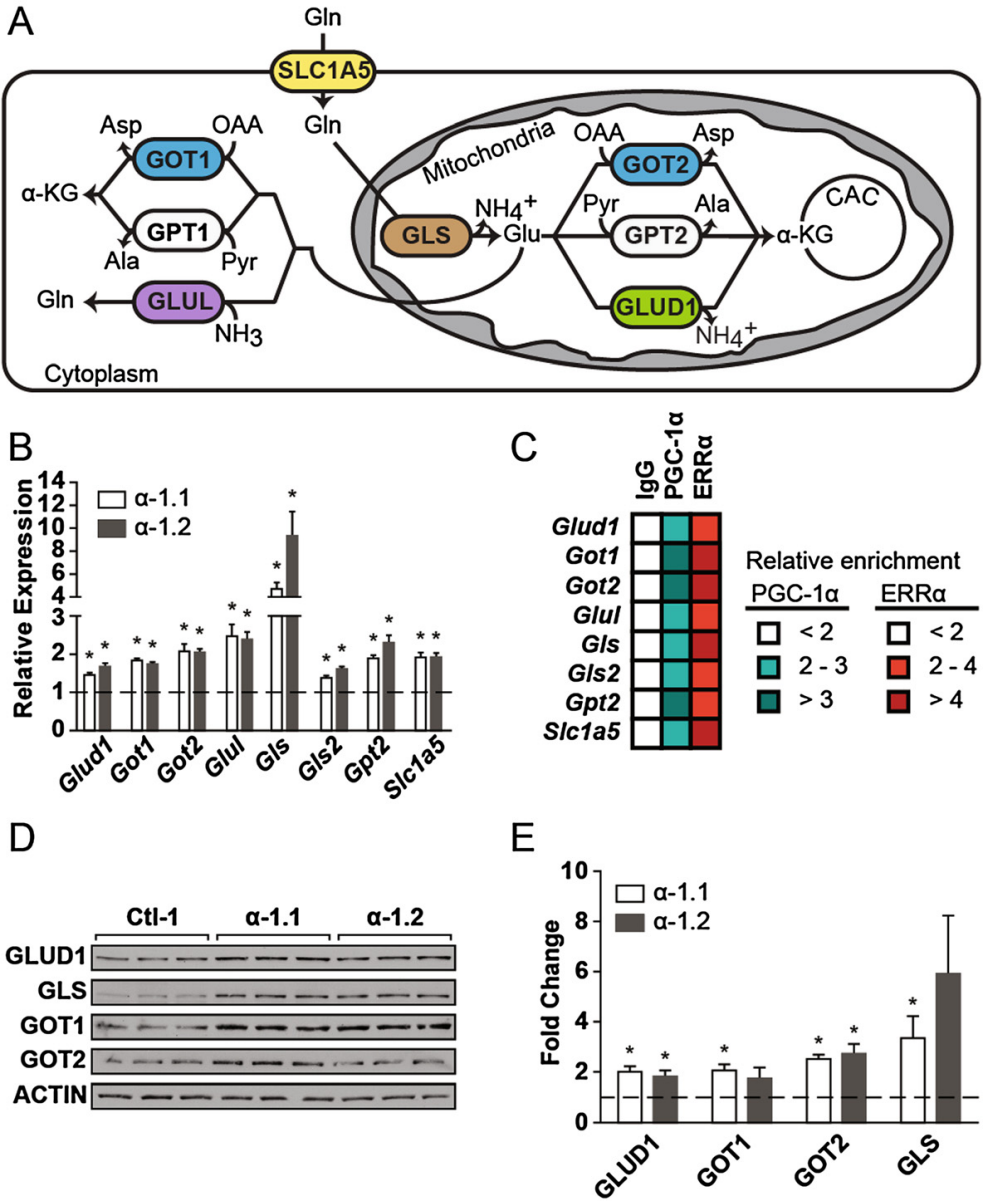
**PGC-1α modulates the expression of glutamine metabolism enzymes. (A)** Schematic representation of glutamine metabolism in cells. SLC1A5, solute carrier family 1, member 5; GLS1, kidney-type glutaminase; GLS2, liver-type glutaminase; GLUD1, glutamate dehydrogenase 1; GOT2, mitochondrial glutamic-oxaloacetic transaminase; GPT2, mitochondrial glutamic pyruvate transaminase; GOT1, cytoplasmic glutamic-oxaloacetic transaminase; GPT1, cytoplasmic glutamic pyruvate transaminase; GLUL, glutamate-ammonia ligase; Gln, glutamine; Glu, glutamate; OAA, oxaloacetate; Asp, aspartate; Pyr, pyruvate; Ala, alanine; α-KG, α-ketoglutarate; CAC, citric acid cycle. **(B)** Expression of glutamine metabolism genes in ERBB2/Neu-induced breast cancer cells (NT2196) with increased expression of PGC-1α (α-1.1, α-1.2) normalized to that in control cells. Data are presented as means ± S.E.M., n = 4. *P <0.05, paired Student's *t*-test. **(C)** ChIP analyses for PGC-1α and ERRα at ERRE sites on the promoter of glutamine metabolism genes in NT2196 cells with increased expression of PGC-1α (α-1.1). Data represent the fold enrichment for two independent experiments. **(D)** Representative western blot for select glutamine metabolism enzymes in NT2196 cells with increased expression of PGC-1α (α-1.1, α-1.2) and control (Ctl-1). **(E)** Quantification of five independent western blot experiments, normalized to ACTIN levels. Data are presented as means ± S.E.M., n = 5. *P <0.05, paired Student's *t*-test.

To assess the impact of PGC-1α on the expression of glutamine metabolism genes, we used clones of ERBB2/Neu-induced breast cancer cells with modestly increased expression of PGC-1α (α-1.1 and α-1.2) and control cells (Ctl-1) [13]. These cells have been extensively characterized [13], and as expected, they display an approximately 30% increase in expression of well-known PGC-1α mitochondrial target genes [13] [see Additional file 3: Figure S1A]. Here we reveal that the mRNA expression of enzymes involved in glutamine catabolism, transport, and synthesis were significantly increased in PGC-1α clones compared with control (Figure 1A,B). In support of this point, cells with reduced expression of PGC-1α displayed lowered expression of glutamine metabolism genes [see Additional file 3: Figure S1B]. In addition, PGC-1α controls these genes in a cancer-independent context, as PGC-1α muscle-specific transgenic animals exhibited increased expression of several glutamine metabolism genes [see Additional file 3: Figure S1C,D]. Given the known functional relationship between PGC-1α and the estrogen-related receptor α (ERRα) in the control of metabolism genes [10], we tested whether PGC-1α could be recruited at promoter sites targeted by ERRα using chromatin immunoprecipitation (ChIP) experiments. ERBB2/Neu-induced breast cancer cells with increased expression of PGC-1α (α-1.1) displayed two-fold enrichment or greater for PGC-1α and ERRα at the promoter of glutamine metabolism genes (Figure 1C), supporting the tight association between the ERRs and PGC-1 s in the control of metabolic genes [10,12]. Finally, we confirmed that the PGC-1α-mediated increase in expression of glutamine metabolism genes resulted in approximately two-fold or greater increase in protein levels (Figure 1D,E). This coordinated augmentation in mRNA and protein expression, in addition to corresponding enrichments observed in ChIP analyses, demonstrates that PGC-1α and ERRα exert a concerted control of glutamine metabolism genes; such pathway control has been shown to have strong biological relevance [21].

### PGC-1α/ERRα modulate forward and reverse glutamine flux through the citric acid cycle

**Figure 2:**
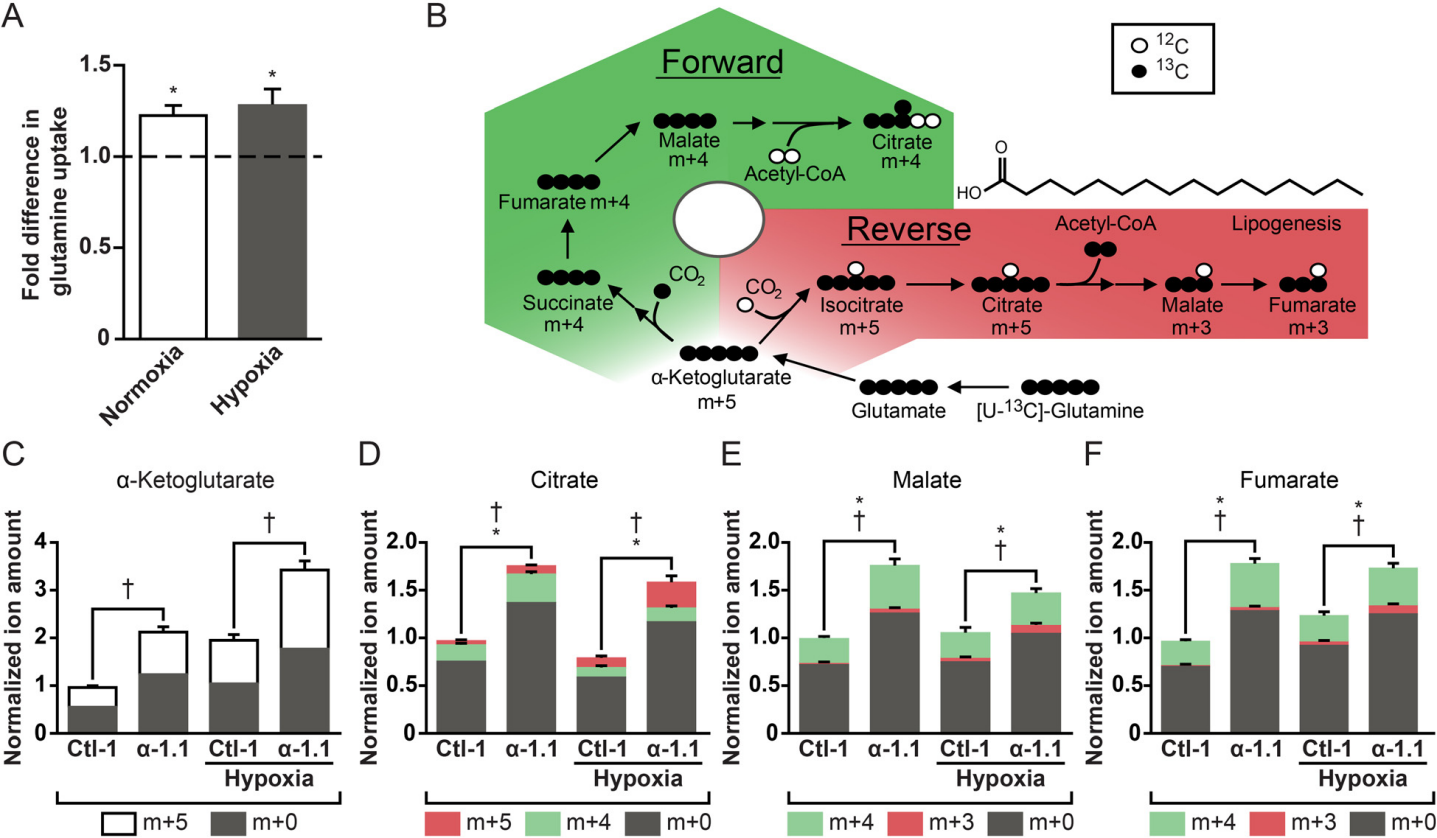
**PGC-1α modulates glutamine metabolism in normoxia and hypoxia. (A)** Glutamine uptake in NT2196 cells with increased expression of PGC-1α (α-1.1) and control. Cells were cultured in either normoxia or hypoxia for 24 h. Data are represented as fold difference for α-1.1 cells compared to control. Data are presented as means ± S.E.M., n = 5. *P <0.05, paired Student's *t*-test. **(B)** Schematic representation depicting metabolomics experiments using [U-^13^C]-glutamine to evaluate forward (green) and reverse (red) citric acid cycle (CAC) fluxes. Herein, ^13^C is represented as black circles and endogenous ^12^C as white circles. **(C)** Mass isotopomer enrichment of α-ketoglutarate in NT2196 cells with increased expression of PGC-1α (α-1.1) and control (Ctl-1) pulsed with [U-^13^C]-glutamine in normoxia and hypoxia. Data are presented as means ± S.E.M., n = 6. †*P* <0.05, paired Student's *t*-test. **(D)** Mass isotopomer enrichment of citrate. Data are presented as means ± S.E.M., n = 6. *(m + 4, forward flux) and †(m + 5, reverse flux) *P* <0.05, paired Student's *t*-test. **(E)** Mass isotopomer enrichment of malate. Data are presented as means ± S.E.M., n = 6. *(m + 4, forward flux) and †(m + 3, reverse flux) *P* <0.05, paired Student's *t*-test. **(F)** Mass isotopomer enrichment of fumarate. Data are presented as means ± S.E.M., n = 6. *(m + 4, forward flux) and †(m + 3, reverse flux) *P* <0.05, paired Student's *t*-test. For panels **C**-**F**, normalized ion amounts were calculated as normalized MDV* multiplied by fold change in steady state levels, relative to Ctl-1 cells in normoxia.

To demonstrate the biological significance of the changes in glutamine gene expression, we first quantified relative glutamine uptake in ERBB2/Neu-induced breast cancer cells with increased expression of PGC-1α (α-1.1) and control cells. The α-1.1 cells showed a 25% increase in glutamine uptake both in normoxia and hypoxia compared with control (Figure 2A). This increase is similar in magnitude to that elicited by oncogenic levels of Myc [3].

To gain insight on the flux of glutamine upon increased PGC-1α expression, α-1.1 and control cells were incubated with [U-^13^C]-glutamine labelled at all five carbons (Figure 2B). α-ketoglutarate m + 5 is generated from glutamine m + 5 ([U-^13^C]-glutamine) and serves as an entry point in the CAC, where it can either generate isocitrate m + 5 by reductive carboxylation (reverse flux) or succinate m + 4 by oxidative decarboxylation (forward flux) (Figure 2B). Reverse CAC flux leads to enrichments in citrate m + 5, malate m + 3, and fumarate m + 3, and forward CAC leads to the enrichment of m + 4 metabolites (Figure 2B). Citrate m + 4 and m + 5 can be used for lipid biosynthesis, but citrate generated specifically through reverse CAC (m + 5) plays a central role in glutamine-derived lipogenesis in cancer cells (Figure 2B) [5-7].

We performed glutamine labeling experiments in normoxia and hypoxia given that reductive carboxylation of glutamine is particularly active in hypoxia [5-7]. In normoxia, α-1.1 cells displayed increased entry of glutamine in the CAC as illustrated by elevated amount of m + 5 α-ketoglutarate compared with control (Figure 2C). The α-ketoglutarate can then enter both the forward and reverse CAC reactions. The α-1.1 cells displayed higher forward and reverse CAC activity compared with controls (Figure 2C-F). The elevated forward flux can be seen from the higher amount of m + 4 citrate, malate, and fumarate, while the elevated reverse flux can be observed in the higher amount of m + 5 citrate as well as m + 3 malate and fumarate (Figure 2D-F) [see Additional file 4: Figure S2A]. The reverse flux represented a small fraction of the total CAC flux in normoxia. In hypoxia, there was an increase in the entry of glutamine in the CAC at α-ketoglutarate both for the control and α-1.1 cells compared with normoxia (Figure 2C). In general, hypoxia caused an increase in the reverse flux for both the control and α-1.1 cells (Figure 2D-F) [see Additional file 4: Figure S2A], but the effects were more pronounced in the α-1.1 cells and can be best appreciated when examining the labeling on citrate (Figure 2D). Indeed, α-1.1 cells displayed a large m + 5 labeling on citrate in hypoxia and devoted a larger fraction of their CAC flux for reverse reactions than control cells in hypoxia (Figure 2D). Together, these metabolomics experiments illustrate that PGC-1α can stimulate glutamine-mediated forward and reverse CAC fluxes in normoxia and hypoxia.

**Figure 3:**
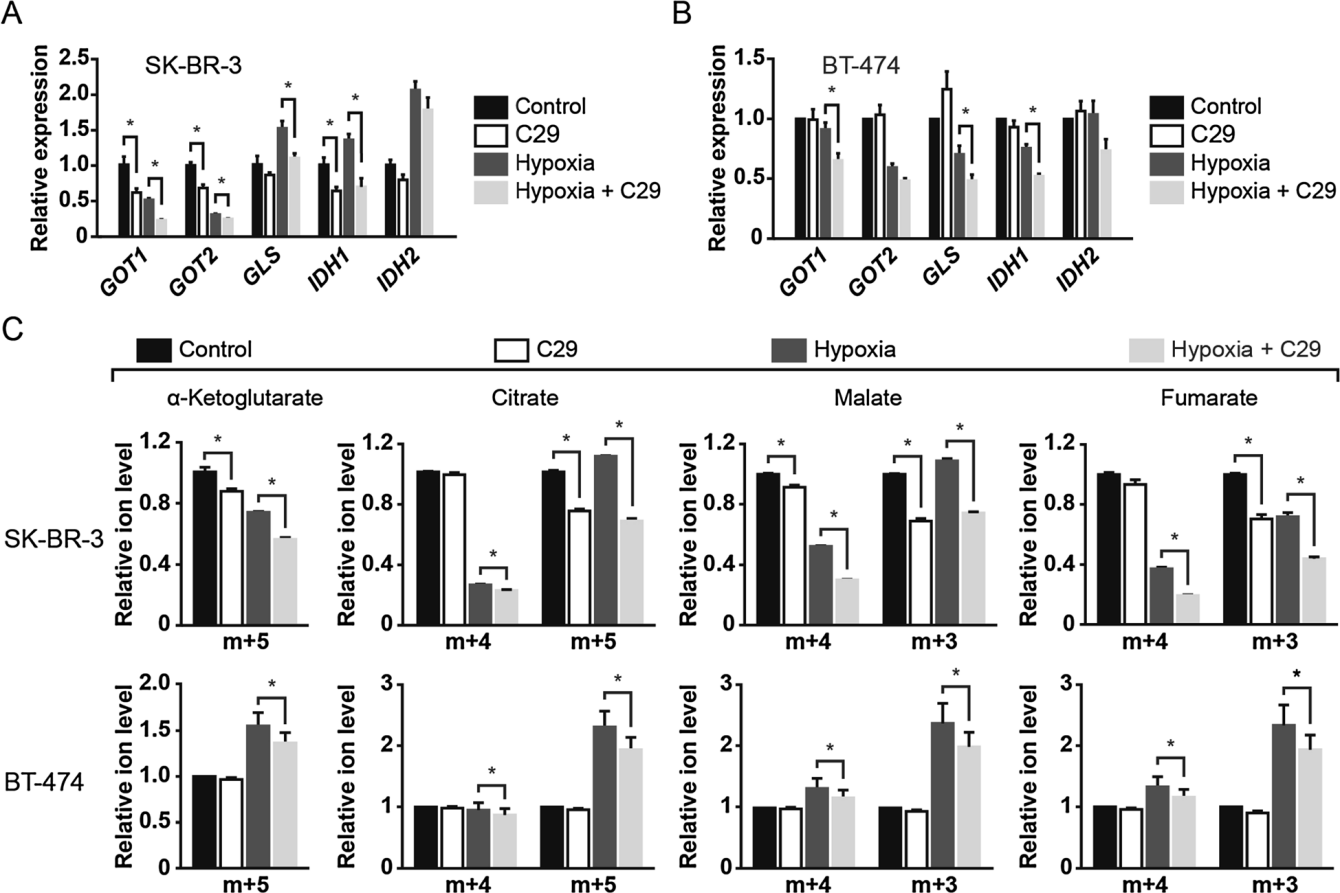
**ERRα modulates glutamine metabolism in normoxia and hypoxia. (A)** Expression of glutamine metabolism genes in SK-BR-3 cells treated with ERRα inhibitor C29 and cultured in either normoxia or hypoxia for 24 h. Expression is normalized to normoxic control cells. Data are represented as means ± S.E.M., from three biological replicates representative of two independent experiments. *P <0.05, unpaired Student's *t*-test. **(B)** Expression of glutamine metabolism genes in BT-474 cells treated with C29 and cultured in either normoxia or hypoxia for 24 h. Expression is normalized to normoxic control cells. Data are represented as means ± S.E.M., from four independent cultures. *P <0.05, paired Student's *t*-test. **(C)** Mass isotopomer distribution analysis of CAC intermediates in SK-BR-3 and BT-474 cells treated as in **A** and **B**, respectively, and pulsed with [U-^13^C]-glutamine. For SK-BR-3, data are represented as means ± S.E.M., from three biological replicates representative of two independent experiments. *P <0.05, unpaired Student's *t*-test. For BT-474, data are represented as means ± S.E.M., from four independent cultures. *P <0.05, paired Student's *t*-test. For panel **C**, relative ion levels represent specific mass isotopomer fractions normalized to that of control cells in normoxia.

Next, we investigated whether ERRα could also regulate glutamine flux through the CAC given that it can be recruited with PGC-1α to the promoter of glutamine metabolism genes. To do so, we used the ERRα pharmacological inhibitor C29 [15] in the ERBB2+ human breast cancer cell lines SK-BR-3 and BT-474. Treatment of SK-BR-3 cells with C29 both in normoxia and hypoxia decreased the expression of glutamine metabolism and CAC genes (Figure 3A). This is supported by a concomitant decrease in glutamine metabolism protein levels [see Additional file 5: Figure S3]. In agreement with these changes, SK-BR-3 cells treated with C29 displayed a decrease in forward CAC flux, most notably in hypoxia, illustrated by reduced fraction of m + 4 citrate, malate, and fumarate (Figure 3C). The reverse CAC flux was significantly decreased in the presence of C29 in normoxia and hypoxia, demonstrated by a reduced fraction of m + 5 α-ketoglutarate and citrate as well as m + 3 malate and fumarate (Figure 3C). In BT-474 cells, C29 decreased the expression of glutamine metabolism and CAC genes and also reduced forward and reverse CAC fluxes in hypoxia (Figure 3B,C). The lack of effect of C29 in BT-474 cells in normoxia is most likely explained by the fact that these cells are not as dependent on ERRα activity as SK-BR-3 cells under standard conditions [22]. Together, the metabolite flux experiments performed with PGC-1α and ERRα illustrate that they both play a central role in controlling glutamine-mediated forward and reverse CAC fluxes in ERBB2+ breast cancer cells. It is important to note that we did not detect glutamine-derived lactate in all ERBB2+ cells we tested [see Additional file 4: Figure S2B-D].

### PGC-1α increases glutamine-mediated fatty acid biosynthesis and favors proliferation in limited nutrient conditions

**Figure 4:**
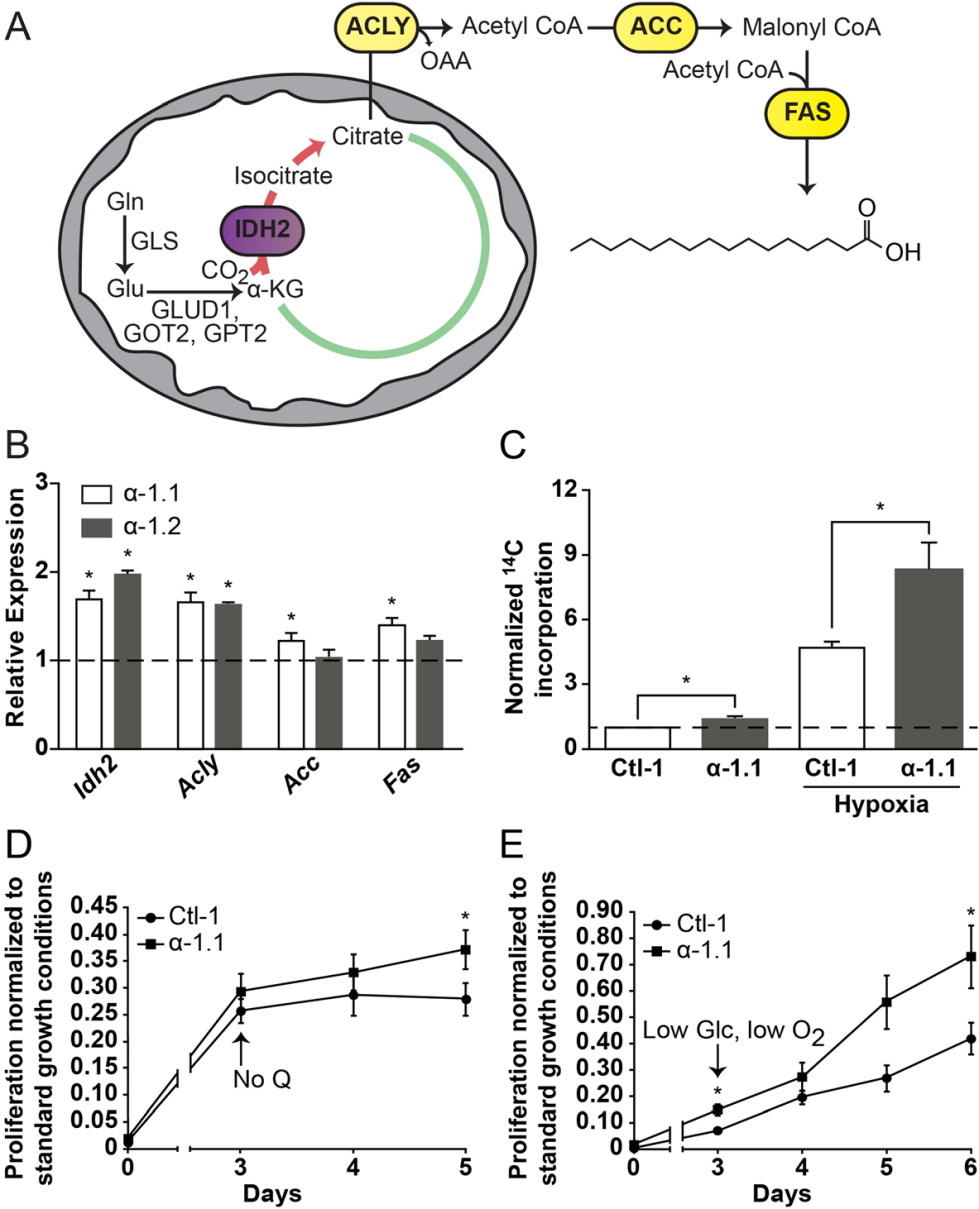
**PGC-1α promotes glutamine-mediated lipid biosynthesis and favors proliferation in limited nutrient conditions. (A)** Schematic representation depicting lipogenesis from glutamine through reverse citric acid cycle (CAC) activity. Glutamine-derived α-ketoglutarate is converted to isocitrate by mitochondrial NADP^+^/NADPH-dependent isocitrate dehydrogenase (IDH2), producing citrate. Citrate is exported to the cytoplasm to generate acetyl CoA and oxaloacetate via ATP citrate lyase (ACLY). Acetyl CoA is converted to malonyl CoA by acetyl CoA carboxylase (ACC). Iterative additions of acetyl CoA to malonyl CoA by fatty acid synthase (FAS) yield fatty acid products (shown: palmitate). Gln, glutamine; Glu, glutamate; OAA, oxaloacetate; GLS1, kidney-type glutaminase; GLS2, liver-type glutaminase; GLUD1, glutamate dehydrogenase 1; GOT2, mitochondrial glutamic-oxaloacetic transaminase; GPT2, mitochondrial glutamic pyruvate transaminase. **(B)** Expression of lipogenic genes in ERBB2/Neu-induced breast cancer cells (NT2196) with increased expression of PGC-1α (α-1.1, α-1.2) normalized to that in control cells. Data are presented as means ± S.E.M., n = 3. *P <0.05, paired Student's *t*-test. **(C)** Incorporation of ^14^C into lipids from trace [U-^14^C]-glutamine in α-1.1 and Ctl-1 cells under normoxia or hypoxia. Counts were normalized for cell number and expressed relative to that of control cells in normoxia. Data are presented as means ± S.E.M., n = 6. *P <0.05, paired Student's *t*-test. **(D)** Relative proliferation of α-1.1 and Ctl-1 cells in the absence of glutamine compared to proliferation in standard glutamine conditions. Data are presented as fold values of cell count in standard glutamine conditions at 5 days ± S.E.M., n = 6. *P <0.05, paired Student's *t*-test. **(E)** Relative proliferation of α-1.1 and Ctl-1 cells in hypoxia under low glucose conditions compared to proliferation in normoxia and standard glucose conditions. Data are presented as fold values of cell count in normoxia and standard glucose conditions at 6 days ± S.E.M., n = 4. *P <0.05, paired Student's *t*-test.

One central function of glutamine metabolism is to replenish the CAC of intermediates that are used for biosynthetic processes, notably citrate that is used in lipid synthesis [2]. Indeed, glutamine has been shown to play a central role in lipid biosynthesis in cancer cells [23,24]. We measured the impact of PGC-1α on the expression of key genes involved in lipid biosynthesis. ERBB2/Neu-induced breast cancer cells with increased expression of PGC-1α (α-1.1 and α-1.2) displayed an elevated expression of lipogenic genes compared with control cells (Ctl-1; Figure 4A,B). In order to determine the involvement of PGC-1α in glutamine-mediated lipid biosynthesis, we incubated α-1.1 cells and control with trace amounts of [U-^14^C]-glutamine and monitored ^14^C incorporation in whole lipid extracts. The α-1.1 cells displayed a significant (48%) increase in ^14^C incorporation compared with controls in normoxia, as well as a robust (63%) increase in ^14^C incorporation compared with controls in hypoxia (Figure 4C). As expected, there was an important increase in glutamine-mediated lipid biosynthesis in hypoxia in both control and α-1.1 cells (Figure 4C) [7]. Also, ERRα is an important regulator of glutamine-mediated lipogenesis as BT-474 cells treated with C29 elicited a significant (62%) decrease in [U-^14^C]-glutamine incorporation into lipids in hypoxia [see Additional file 6: Figure S4A]. These data illustrate that PGC-1α and ERRα are key regulators of glutamine-mediated lipid biosynthesis.

Next, we tested the impact of PGC-1α on the sensitivity of ERBB2/Neu-induced breast cancer cells to glutamine deprivation, low glucose, and hypoxia, in order to mimic the environment of solid growing tumors. The growth of both ERBB2/Neu-induced breast cancer cells with increased expression of PGC-1α (α-1.1) and controls was ablated in the absence of glutamine (Figure 4D), illustrating that ERBB2/Neu-induced breast cancer cells require glutamine for their growth. A similar dependency on glutamine was observed with human ERBB2+ breast cancer cell lines [see Additional file 6: Figure S4B,C]. We then tested the impact of PGC-1α on cellular proliferation in low glucose and hypoxia, two conditions that can favor glutamine usage (Figure 4E) [5,7,25]. The proliferation of α-1.1 cells was less sensitive to a combination of low glucose and hypoxia than controls as indicated by their smaller reduction in proliferation (Figure 4E). Together, these data demonstrate that glutamine is a key metabolite for the growth of ERBB2/Neu-induced breast cancer cells, and that the PGC-1α-mediated increase in glutamine metabolism is associated with protection against low glucose conditions and hypoxia, two conditions that can favor glutamine metabolism.

### Gene expression analyses of PGC-1α and glutamine enzymes in breast cancer patients

**Figure 5:**
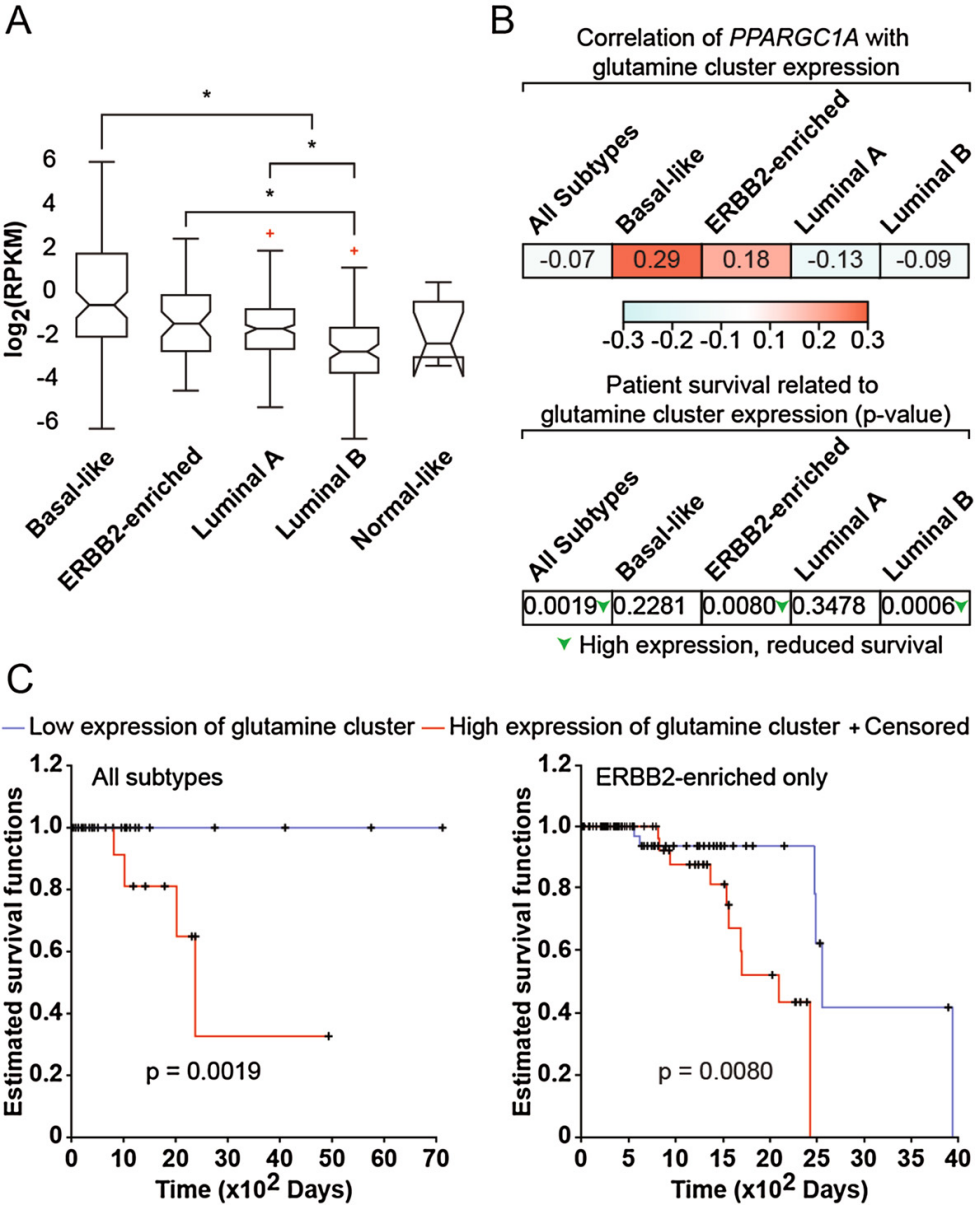
**Expression analyses of PGC-1α and glutamine pathway in breast cancer patients. (A)** Expression of PGC-1α across the different breast cancer clinical subtypes in The Cancer Genome Atlas (TCGA) data set, where crosses represent outliers for this subtype. Expression is given in log2-normalized reads per kilobase of exon model per million mapped reads (RPKM). The number of samples for Basal-like, ERBB2-enriched, Luminal A, Luminal B, and Normal-like is 87, 53, 201, 110, and 7, respectively. PGC-1α expression is higher in basal than luminal A and B subtypes, higher in luminal A subtype than luminal B subtype, and higher in ERBB2-enriched than luminal B subtype. *P* <1E-13 for Kruskal-Wallis, *P <0.05 using *a posteriori* Tukey-Kramer test. **(B)** (top) Expression of the glutamine metabolism genes is correlated with that of PGC-1α. The table shows the Pearson correlation coefficients between PGC-1α expression and the pathway score of the glutamine cluster. Colors represent the intensity of correlation as per the gradient shown. (bottom) Patient survival is related to glutamine cluster expression. The table shows *P* values for survival. Higher expression of glutamine cluster correlated with reduced survival in ERBB2-enriched and luminal B subtypes as well as in combined subtypes. Green arrows represent significant differences. *P* <0.05, logrank test. **(C)** High expression of glutamine metabolism genes is associated with reduced survival in all patients (all subtypes, left) and ERBB2-enriched patients (right). Kaplan-Meier survival curves represent the differences in survival between patients with high and low expression of the glutamine cluster.

We examined the expression of PGC-1α (*PPARGC1A*) and glutamine enzymes in 732 breast cancer patients from The Cancer Genome Atlas (TCGA) to determine their expression pattern across breast cancer subtypes and correlation with survival. *PPARGC1A* expression was highest in basal and ERBB2-enriched breast cancer subtypes, which have poorer prognosis than luminal A and B subtypes (Figure 5A). The expression of glutamine enzymes (glutamine cluster) was positively correlated with *PPARGC1A* expression in the basal and ERBB2-enriched breast cancer subtypes, in agreement with the fact that these subtypes have the highest expression of *PPARGC1A* (Figure 5B). Then, we determined if the expression of the glutamine cluster was correlated with clinical outcome. High expression of the glutamine cluster was associated with reduced survival in ERBB2-enriched and luminal B subtypes, and this association held true when looking at all breast cancer patients (Figure 5B,C). The fact that high expression of the glutamine cluster is associated with reduced survival in luminal B subtype but is not positively correlated with *PPARGC1A* expression suggests that, while glutamine metabolism is clinically relevant in this subtype, factors other than PGC-1α are driving the expression of this pathway. Overall, these data provide clinical relevance to the notion that PGC-1α is a central regulator of glutamine metabolism in ERBB2+ breast cancer cells.

## Discussion

In this manuscript, we report that the PGC-1α/ERRα axis is a key regulator of glutamine metabolism in ERBB2+ breast cancer cells. We revealed that PGC-1α/ERRα bind the promoter of glutamine metabolism genes and regulate their expression, which ultimately controls the flux of glutamine in cells. This can contribute to increased lipid biosynthesis, especially under hypoxic conditions. Finally, we demonstrate that the PGC-1α-mediated control of glutamine metabolism has clinical relevance in ERBB2+ breast cancer patients.

Even though the importance of glutamine metabolism in cancer cells is well appreciated, much less is known regarding central regulators of glutamine metabolic pathways. Major transcriptional regulators of glutamine metabolism identified so far are c-Myc [3,4,26,27] and HIF-1 [5,7]. In this study, we reveal that PGC-1α/ERRα are central regulators of glutamine-mediated forward and reverse CAC fluxes. Interestingly, a recent report showed that glutamine reductive carboxylation for lipogenesis is particularly active in brown fat cells [28], the same cell type where PGC-1α was discovered and which display very high expression of this coactivator [29]. In recent years, PGC-1s/ERRs have been involved in the control of the expression of numerous metabolic pathways in breast cancer cells [12,22,30]. Both PGC-1α and ERRα have been shown to promote the growth of ERBB2+ tumors *in vivo* and to regulate the metabolism of this specific breast cancer subtype [12,13,22,31]. Interestingly, increased expression of PGC-1α reduced the proliferation of ERBB2+ cells *in vitro*, but promoted tumor growth *in vivo* by promoting nutrient supply [13]. We show here that ERBB2+ cells are dependent on glutamine for their growth as complete glutamine removal from cell culture severely compromised cell growth. Although the glucose dependence of ERBB2+ cells is well known [32], the role of glutamine in this system has been largely overlooked. A point supporting the importance of glutamine in ERBB2+ tumors is the observation that ERBB2 signaling elevates c-Myc expression, which is a central regulator of glutamine metabolism [22]. Furthermore, it would not be surprising that the pro-growth effect of PGC-1α and ERRα in ERBB2+ tumors is mediated at least in part through their positive impact on glutamine metabolism. In support of this point, the expression of PGC-1α and that of the glutamine pathway are positively correlated in ERBB2+ breast cancer patients, and high expression of glutamine metabolism enzymes is associated with shorter survival. It is important to emphasize that we do not exclude the possibility that PGC-1α may associate with other transcription factors, in addition to ERRα, to control the expression of glutamine metabolism genes.

## Conclusions

In this paper, we reveal that the PGC-1α/ERRα axis is a central regulator of glutamine metabolism in ERBB2+ breast cancer. PGC-1α/ERRα likely regulate similar metabolic programs in diverse cancer types as the metabolic targets of PGC-1α/ERRα in various normal tissues and cancer cells/tumors are similar. The positive or negative impact of PGC-1α/ERRα on tumor growth will depend on the specific metabolic alterations of each tumor. There is significant effort currently underway to associate metabolic signatures to specific oncogenes. Furthermore, it is important to appreciate that the PGC-1α/ERRα axis also regulates the expression of genes not directly associated with metabolism; these genes may be equally or even more important in defining the roles of PGC-1α/ERRα in various cancer types. For example, ERRα regulates the expression of genes located in the ERBB2 amplicon that are involved in signal transduction, cell migration and invasion, as well as tamoxifen resistance [31]. Overall, much remains to be discovered regarding the importance of PGC-1α/ERRα in contributing to the altered metabolism of various cancer types and to link this new metabolic state with clinical outcome. Also, the importance of PGC-1α/ERRα target genes involved in other hallmarks of cancer cells such as migration/invasion, apoptosis, and angiogenesis [1] needs to be established. That being said, the identification of PGC-1α/ERRα as central regulators of glutamine metabolism may open novel therapeutic avenues for tumors showing strong dependence on this metabolic pathway.

## Abbreviations

CAC: Citric acid cycle; ChIP: Chromatin immunoprecipitation; RPKM: Reads per kilobase of exon model per million mapped reads; TCGA: The Cancer Genome Atlas.

## Competing interests

The authors declare that they have no competing interests.

## Authors’ contributions

JSP, RGJ, VG, SM and SPG designed the experiments; SM and SPG performed qPCR, immunoblotting, and protein quantification; GD performed ChIP analyses; SPG performed GC/MS and glutamine uptake experiments, with technical assistance and counseling by DA; SPG and SM performed mass isotopomer distribution analysis; SPG and DJP performed siRNA experiments; SM and BF performed ^14^C-lipid incorporation assays; SM performed proliferation experiments; AW and KH performed preliminary metabolomics experiments; UDA performed bioinformatics analyses; JSP, SM and SPG wrote the paper. All authors read and approved the final manuscript.

## Authors’ information

Co-first authors: Shawn McGuirk and Simon-Pierre Gravel.

## Supplementary Material

Additional file 1 Supplementary methods. Click here for file

Additional file 2: Table S1 GC/MS metabolites and fragments used for mass isotopomer distribution analysis. Click here for file

Additional file 3: Figure S1 PGC-1α regulates the expression of key mitochondrial and glutamine metabolism enzymes. **(A)** Expression of mitochondrial genes in ERBB2/Neu-induced breast cancer cells (NT2196) with increased expression of PGC-1α (α-1.1, α-1.2) normalized to that of control cells. Data are presented as means ± S.E.M., n = 6. *P <0.05, paired Student's *t*-test. **(B)** Expression of glutamine genes in BT-474 cells treated with siPGC-1α for 120 h normalized to that of cells treated with control siRNA. Data are presented as means ± S.E.M., n = 4. *P <0.05, paired Student's *t*-test. **(C)** Expression of glutamine metabolism genes in mck-PGC-1α transgenic mice normalized to that of wild-type mice. Data are presented as means ± S.E.M., n = 6. *P <0.05, paired Student's *t*-test. **(D)** Representative western blot of selected glutamine metabolism enzymes in wild-type and mck-PGC-1α transgenic mice. Click here for file

Additional file 4: Figure S2 PGC-1α increases glutamine flux to malate and fumarate, and ERBB2+ breast cancer cell lines lack glutamine-derived pyruvate and lactate. **(A)** Mass isotopomer enrichment of malate and fumarate. Data are presented as means ± S.E.M., n = 6. *P <0.05, paired Student's *t*-test. **(B)** Mass isotopomer distribution analysis of pyruvate (left) and lactate (right) in ERBB2/Neu-induced breast cancer cells (NT2196) with increased expression of PGC-1α (α-1.1) and Control (Ctl-1) under normoxia or hypoxia, and pulsed with [U-^13^C]-glutamine. Data are presented as means ± S.E.M., n = 3. **(C)** Mass isotopomer distribution analysis of pyruvate (left) and lactate (right) in SK-BR-3 cells under normoxia or hypoxia. Cells were pulsed with [U-^13^C]-glutamine. Data are presented as means ± S.E.M., n = 3. **(D)** Mass isotopomer distribution analysis of pyruvate (left) and lactate (right) in BT-474 cells under normoxia or hypoxia. Cells were pulsed with [U-^13^C]-glutamine. Data are presented as means ± S.E.M., n = 3. For panel **A**, specific ion amounts are from Figure 2E-F and were normalized to that of Ctl-1 cells in normoxia. For panels **B**-**D**, relative ion levels represent specific mass isotopomer fractions normalized to that of control cells in normoxia. Click here for file

Additional file 5: Figure S3 ERRα modulates the expression of glutamine metabolism enzymes **(A)** Representative western blot for ERRα and select glutamine metabolism enzymes in SK-BR-3 cells treated with ERRα inhibitor C29 and cultured in either normoxia or hypoxia for 24 h **(B)** Quantification of five independent western blot experiments, normalized to ACTIN levels. Data are presented as means of C29-treated SK-BR-3 cells relative to control (DMSO) ± S.E.M., n = 5. *P <0.05, paired Student's *t*-test. Click here for file

Additional file 6: Figure S4 ERRα promotes glutamine-mediated lipogenesis, and glutamine deprivation limits the proliferation of human ERBB2+ breast cancer cell lines. **(A)** Incorporation of ^14^C into lipids from trace [U-^14^C]-glutamine in BT-474 cells under normoxia or hypoxia treated with C29 or DMSO control. Counts were normalized for cell number and expressed relative to the count of control cells in normoxia. Data are presented as means ± S.E.M., n = 4. *P <0.05, paired Student's *t*-test. **(B)** Proliferation of BT-474 cells in the presence or absence of glutamine. Data are presented as means ± S.E.M., n = 3. **(C)** Proliferation of SK-BR-3 cells in the presence or absence of glutamine. Data are presented as means ± S.E.M., n = 3. Click here for file
