# Supplementary material for: PGC-1α supports glutamine metabolism in breast cancer
Source: Cancer Metab. 2013 Dec 5;1:22. doi: 10.1186/2049-3002-1-22 (PMC4178216; doi:10.1186/2049-3002-1-22)
Supplement: Additional file 1 — Supplementary methods. [file 2049-3002-1-22-S1.doc]

**Supplementary Methods**

**In vitro cell proliferation assay**

For the proliferation experiments testing the impact of glutamine deprivation and hypoxia, the cells were seeded at a density of 20,000 cells per 35mm plate. For the proliferation experiments testing the impact of low glucose, the cells were seeded at a density of 7,500 per 35mm plate. For the proliferation experiments testing the impact of glutamine deprivation, the cells were grown in glutamine-free DMEM supplemented with 10% dialyzed FBS, 10 μg/mL insulin, 20 mM HEPES, penicillin/streptomycin, 1 μg/mL puromycin, 400 μg/mL G418 as well as glutamine (for the 4 mM glutamine condition), at 37°C and 5% CO_2_. For the proliferation experiments testing the impact of low glucose, the cells were grown in glucose-free DMEM supplemented with 10% dialyzed FBS, 10 μg/mL insulin, 20 mM HEPES, penicillin/streptomycin, 1 μg/mL puromycin, 400 μg/mL G418 as well as glucose (25 mM or 1 mM), at 37°C and 5% CO_2_. For the proliferation experiments testing the impact of hypoxia, cells were grown first in standard DMEM media and subsequently in glucose-free DMEM media with dialyzed serum and supplemented with 25 mM glucose and incubated in normoxia (21% O_2_) or supplemented with 1 mM glucose and incubated in hypoxia (1% O_2_). To determine cell counts, cells were washed, trypsinized, and counted using a TC10 automated cell counter (Bio-Rad). Viability was determined by exclusion of trypan blue dye.

**Mass isotopomer distribution analysis**

ERBB2/Neu-induced breast cancer cells (NT2196) ectopically expressing PGC-1α (α-1.1) and Control (Ctl-1) were grown to 50% to 60% confluence in 60mm plates. The medium was aspirated and discarded. The plates were washed with PBS. Growth media were replaced with 2 mL of DMEM high glucose lacking glutamine supplemented with 10% dialyzed FBS (Wisent), 10 μg/mL insulin, 20 mM HEPES and 2 mM [U-^13^C]-glutamine (Cambridge Isotope Laboratories). After an incubation period of 15 min, media were removed, plates were set on ice, and cells were immediately washed 3 times with 2 mL ice-cold normal saline solution (NaCl, 9 g/L). BT-474 and SK-BR-3 cells were treated similarly, but with a 30 min exposure to media containing 2 mM [U-^13^C]-glutamine. Cellular metabolism was then quenched by the addition of 400 μL of 80%(v/v) methanol at -80˚C. Cell suspensions were rapidly scraped and transferred to pre-chilled 1.5 mL tubes on ice. This process was repeated to ensure good sample recovery. Suspensions were then subjected to sonication using a BioRuptor (UCD-200 TM, Diagenode) for 10 min (30 sec ON, 30 sec OFF) at the higher setting. Cellular debris were cleared by centrifugation at 21,000 x g at 4˚C for 10 min. Supernatants were stored at -80˚C until subsequent treatments. 1 µL of internal standard myristic acid-D_27_ dissolved in pyridine (750 ng/µL) was added to each sample, which were subsequently dried by vacuum centrifuge (Labconco, Kansas City, MO, USA) (≤ 4˚C, 18-24h). Pellets were resuspended in 30 μL pyridine containing 10 mg/mL methoxyamine hydrochloride (Sigma), vortexed, and sonicated to ensure dissolution. Samples were transferred to sealed autoinjection vials and heated at 70˚C for 30 min. 70 μL of *N*-*tert*-Butyldimethylsilyl-*N*-methyltrifluoroacetamide (MTBSTFA) (Sigma) was added and vials were incubated for 1h at 70˚C.

GC/MS analysis was performed using an Agilent 5975C GC/MS equipped with a DB-5MS+DG (30 m x 250 µm x 0.25 µm) capillary column (Agilent J&W, Santa Clara, CA, USA). All data were collected by electron impact set at 70 eV. 1 μL of the derivatized sample was injected in the GC in splitless mode with inlet temperature set to 280˚C, using helium as a carrier gas with a flow rate of 1.5512 mL/min (rate at which myristic acid elutes at 17.94 min). The quadrupole was set at 150˚C and the GC/MS interface at 285˚C. The oven program for all metabolite analyses started at 60˚C held for 1 min, then increasing at a rate of 10˚C/min until 320˚C. Bake-out was at 320˚C for 10 min. Sample data were acquired both in scan (1-600 m/z) and selected ion monitoring (SIM) modes. All metabolites used in this study were previously validated using authentic standards (all standards from Sigma) to confirm mass spectra and retention times. Integration of ion intensities was done using the Agilent ChemStation software. [U-^13^C]-glutamine flux analysis was achieved by mass isotopomer distribution analysis using an in-house algorithm adapted from . The atomic composition of the TBDMS-derivatized metabolite fragments (M-57) was determined (molecular formulas in Additional file 2; Table S1), and, using the algorithm, matrices correcting for natural contribution of isotopomer enrichment were generated for each metabolite (C_total_^-1^). The mass distribution of each metabolite was normalized (such that the sum of the distribution equals 1) and rearranged in vector form; the resulting mass distribution vector (MDV) was multiplied into the appropriate correction matrix (MDV* = C_total_^-1^ ⋅ MDV). The corrected mass distribution vector (MDV*) was again normalized to 1. These values correspond to the proportional metabolite flux in the cell, and ion level values are expressed as fractions of the total pool of the particular metabolite examined. This methodology was applied for proportional flux analysis in SK-BR-3 and BT-474 cells. To take into account that Ctl-1 and α-1.1 differ in cell size and metabolite pool size, a different methodology was used to evaluate flux. First, the steady state level of each metabolite was determined by summing up all integrated ion intensities for a given metabolite and dividing this value by the integrated ion intensities of the internal standard, myristic acid D_27_. This corrected value was subsequently divided by the mean cell number determined from 2 to 3 culture plates grown in parallel to the experimental “flux” plates. The steady state level of a metabolite is presented as a fold change between cell lines and treatments, with normoxic Ctl-1 set to 1. Normalized ion amounts were calculated as normalized MDV* multiplied by fold change in steady state levels.

**Mouse real-time qPCR primers**

*Acc,* GTCCCCAGGGATGAACCAATA, GCCATGCTCAACCAAAGTAGC ; *Acly,* AAGCCTTTGACAGCGGCATCATTC, TTGAGGATCTGCACTCGCATGTCT ; *Cs*, ATCCATAGTGACCATGAGGG, TCAGTGCCTCAGATACAGTTT; *Idh2,* AACACCGACGAGTCCATTT, CTGATAAGGTCCTGGTTCCC; *Fas,* GGAGGTGGTGATAGCCGGTAT, TGGGTAATCCATAGAGCCCAG ; *Fh1*, TTGGACAGGAATTCAGTGGT, ATCGCTTCACACTGAGTAGG; *Glud1,* GGTCCCCACAGCAGAGTTCCAGGA, CACACCAGCTTCATTGTACACCTT; *Glul,* ACTGCGCTGCAAGACCCGTA, GTTGGTCTCTGCAGGTTTCCGG; *Gls,* GGGCATGATGTGTTGGTCTCCT, GCTGACAGAGCAAACCTTCGG; *Gls2,* TTTGCTGCATATAGTGGAGATGTC, GTTGAACTGCACAGCATCGTCCAG; *Got1,* CCTATTGCTACTGGGATGCGG, CCAGATGCAAAGCCCTGATAG; *Got2*, TCGCAGCAACCATCCTGACTTCTC, AGAACTCCTTGGTCAGCCGCTC; *Gpt1,* CTGCGCCAGGGTGTGAAGAA, GCATGCCTGCAAGATGCGTT; *Gpt2,* AGAAGATCTGTTTAACCAAGTCCC, CCTCCAGGAGCTTCATGCAGTAGA; *Mdh2*, CCAGATTGCCTCAAAGGTTG, TTCGGGGTACACTGAGAGAT; *Ndufb5,* TGGCAAGAGACTGTTTGTCG, AGCTCGGCTTCACCAATAAA; *Slc1a5,* ACCATGGTCCTGGTCTCCTG, GCCAGTCCACGGCCAAGATC ; *Sod2,* GCCTGCACTGAAGTTCAATG, ATCTGTAAGCGACCTTGCTC; *Tbp,* ACCTTATGCTCAGGGCTTGG, GCCATAAGGCATCATTGGAC ; *Ucp2,* CAGGTCACTGTGCCCTTACCAT, CACTACGTTCCAGGATCCCAAG;

**Human real-time qPCR primers**

*B2M,* GTGCTCGCGCTACTCTCTC, GTCAACTTCAATGTCGGAT; *CS*, CAACTCAGGACGGGTTGTTCCAGG, GTAGTAATTCATCTCCGTCATGCC; *ESRRA,* GCTGCCCTGCTGCAACTAGTG, GCCGCCGCTCAGCACCCCCTC ; *FH*, CCATGTTGCTGTCACTGTCGGAGG, CATACCCTATATGAGGATTGAGAG; *GLS,* GGTCTCCTCCTCTGGATAAGATGG, CCCGTTGTCAGAATCTCCTTGAGG; *GLS2*, GGCAGAGAGACGCCACACAG, AGTGGCCTTTAGTGCAGTGGT; *GLUL*, CTCGCGGCCTAGCTTTACCC, CCACTCAGGCAACTCTTCCACA; *GOT1,* ACTGCCGACTTCAGGGAGGA, GCACACCTCCTACCCGCTTC; *GOT2,* CTGGGAGTTGGTGCCTACCG, CTGCACAGTGACAAACCGGC; *IDH1,* ACCAATCCCATTGCTTCCATTTTT, TCAAGTTTTCTCCAAGTTTATCCA; *IDH2,* CAGGAGATCTTTGACAAGCAC, ATGAGGTCTTGGTTCCCATC; *MDH2*, GCTCTGCCACCCTCTCCATG, TTTGCCGATGCCCAGGTTCTTCTC; *PPARGC1A*, CCTGTGATGCTTTTGCTGCTCTTG, AAACTATCAAAATCCAGAGAGTCA; *PPARGC1B*, GTACATTCAAAATCTCTCCAGCGACATG, GAGGGCTCGTTGCGCTTCCTCAGGGCA; *SLC1A5*, CAAGGAGGTGCTCGATTCGT, ACCCTGGTTCCGGTGATATTC ;

**ChIP-quantification primers**

*Asct2,* GATCCCCCTAAGGCTGACCCCAAAGG, GCAACCACCTGCTTTCGCACTCTGGT; *Glud1,* CAAGCTGGTGGAAGACCTGAAGACCC, GAAGGAGAGGCTCAACACATGGTTGC; *Glul,* GCGCAGCTCACTACGGAGGCAGAAAGCT, TCTTTTGAAGAGAGGACCCACCAACGGC; *Gls,* AGGCTGAGCCAATGGAAGAGCTTGCA, GCCTGCAGCCAGCCGAAGTTGCTCTG; *Gls2,* CAAACCAAGACCACACCTTTAATCCC, AACACAAAACATATACGTGCTCCCCC; *Got1,* CTAGTATGTGCTCATCCCTTACTTTCTGC, CAAGTGTCTGGCACAGGACTCTAGTGGTC; *Got2,* GCTTCCAGTTAATCATCCGCTACGC, GGACATGAAAGCATTCTGAACCTTG; *Gpt2,* CGAATACAGGGAGCCAGGAGGTGCTG, AGCTGCGCCTAATGTGGGTATGCAAC;

**Supplementary References**

1. Nanchen A, Fuhrer T, Sauer U: **Determination of metabolic flux ratios from 13C-experiments and gas chromatography-mass spectrometry data: protocol and principles.** *Methods Mol Biol* 2007, **358:**177-197.

2. Bhalla K, Hwang BJ, Dewi RE, Ou L, Twaddel W, Fang HB, Vafai SB, Vazquez F, Puigserver P, Boros L, Girnun GD: **PGC1alpha promotes tumor growth by inducing gene expression programs supporting lipogenesis.** *Cancer Res* 2011, **71:**6888-6898.

3. Dolinska M, Dybel A, Hilgier W, Zielinska M, Zablocka B, Buzanska L, Albrecht J: **Glutamine transport in C6 glioma cells: substrate specificity and modulation in a glutamine deprived culture medium.** *J Neurosci Res* 2001, **66:**959-966.

4. Klimcakova E, Chenard V, McGuirk S, Germain D, Avizonis D, Muller WJ, St-Pierre J: **PGC-1alpha promotes the growth of ErbB2/Neu-induced mammary tumors by regulating nutrient supply.** *Cancer Res* 2012, **72:**1538-1546.

5. Deblois G, Hall JA, Perry MC, Laganiere J, Ghahremani M, Park M, Hallett M, Giguere V: **Genome-wide identification of direct target genes implicates estrogen-related receptor alpha as a determinant of breast cancer heterogeneity.** *Cancer Res* 2009, **69:**6149-6157.

6. Wasa M, Wang HS, Okada A: **Characterization of L-glutamine transport by a human neuroblastoma cell line.** *Am J Physiol Cell Physiol* 2002, **282:**C1246-1253.
