# Supplementary material for: PGC-1α supports glutamine metabolism in breast cancer
Source: Cancer Metab. 2013 Dec 5;1:22. doi: 10.1186/2049-3002-1-22 (PMC4178216; doi:10.1186/2049-3002-1-22)
Supplement: Additional file 2: Table S1 — GC/MS metabolites and fragments used for mass isotopomer distribution analysis. [file 2049-3002-1-22-S2.pdf]

Table S1: GC/MS metabolites and fragments used for mass isotopomer distribution analysis

Metabolite	Formula (M-57)	Quantifying ions	Qualifying ion	Typical Rt (min)
Myristic acid-D_{27}	$C_{16}D_{27}H_6O_2Si$	312	132	17.936
Citrate	$C_{20}H_{39}O_6Si_3$	459-465	431	22.366
α-Ketoglutarate oxime	$C_{14}H_{28}N_1O_5Si_2$	346-351	258	17.292
Fumarate	$C_{12}H_{23}O_4Si_2$	287-291	329	15.178
Malate	$C_{18}H_{39}O_5Si_3$	419-423	287	18.203
Pyruvate oxime	$C_6H_{12}N_1O_3Si$	174-177	115	8.941
Lactate	$C_{11}H_{25}O_3Si_2$	261-264	233	11.733
