# Supplementary figures and images for: PGC-1α supports glutamine metabolism in breast cancer
Source: Cancer Metab. 2013 Dec 5;1:22. doi: 10.1186/2049-3002-1-22 (PMC4178216; doi:10.1186/2049-3002-1-22)

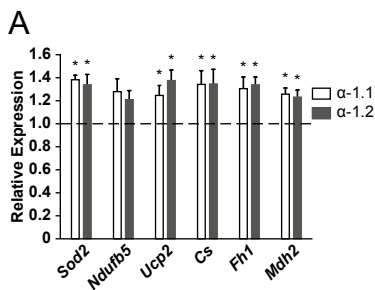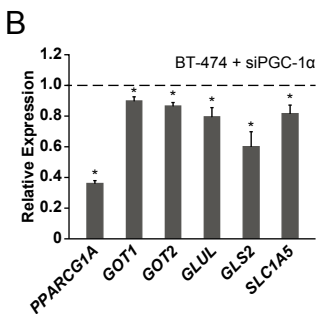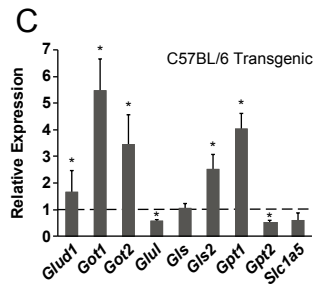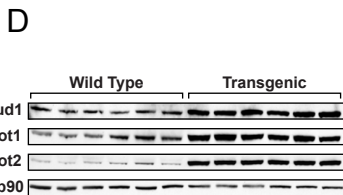

Supplement: Additional file 3: Figure S1 — PGC-1α regulates the expression of key mitochondrial and glutamine metabolism enzymes. (A) Expression of mitochondrial genes in ERBB2/Neu-induced breast cancer cells (NT2196) with increased expression of PGC-1α (α-1.1, α-1.2) normalized to that of control cells. Data are presented as means ± S.E.M., n = 6. *P <0.05, paired Student's t-test. (B) Expression of glutamine genes in BT-474 cells treated with siPGC-1α for 120 h normalized to that of cells treated with control siRNA. Data are presented as means ± S.E.M., n = 4. *P <0.05, paired Student's t-test. (C) Expression of glutamine metabolism genes in mck-PGC-1α transgenic mice normalized to that of wild-type mice. Data are presented as means ± S.E.M., n = 6. *P <0.05, paired Student's t-test. (D) Representative western blot of selected glutamine metabolism enzymes in wild-type and mck-PGC-1α transgenic mice. [file 2049-3002-1-22-S3.pdf]

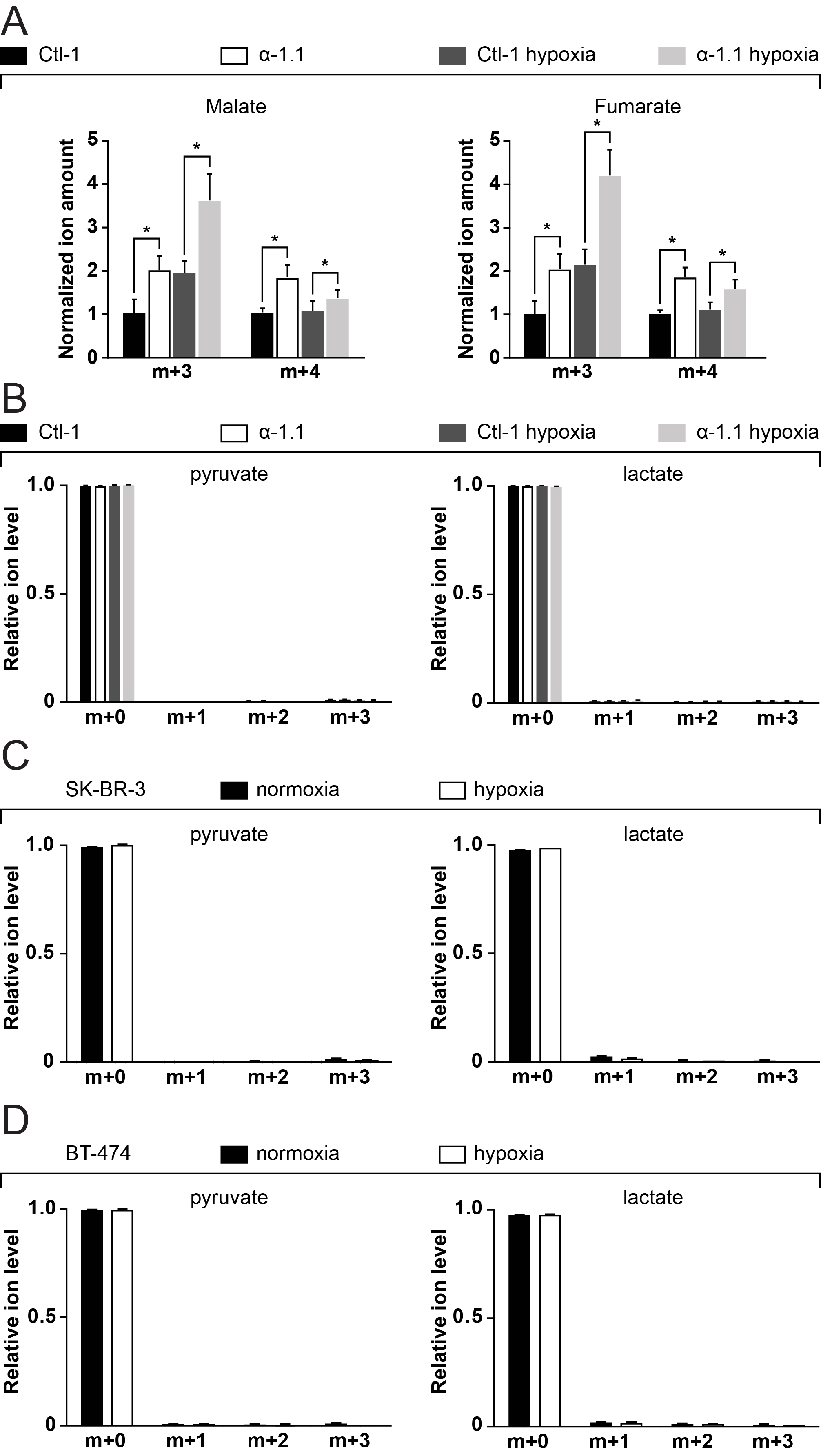

Supplement: Additional file 4: Figure S2 — PGC-1α increases glutamine flux to malate and fumarate, and ERBB2+ breast cancer cell lines lack glutamine-derived pyruvate and lactate. (A) Mass isotopomer enrichment of malate and fumarate. Data are presented as means ± S.E.M., n = 6. *P <0.05, paired Student's t-test. (B) Mass isotopomer distribution analysis of pyruvate (left) and lactate (right) in ERBB2/Neu-induced breast cancer cells (NT2196) with increased expression of PGC-1α (α-1.1) and Control (Ctl-1) under normoxia or hypoxia, and pulsed with [U-13C]-glutamine. Data are presented as means ± S.E.M., n = 3. (C) Mass isotopomer distribution analysis of pyruvate (left) and lactate (right) in SK-BR-3 cells under normoxia or hypoxia. Cells were pulsed with [U-13C]-glutamine. Data are presented as means ± S.E.M., n = 3. (D) Mass isotopomer distribution analysis of pyruvate (left) and lactate (right) in BT-474 cells under normoxia or hypoxia. Cells were pulsed with [U-13C]-glutamine. Data are presented as means ± S.E.M., n = 3. For panel A, specific ion amounts are from Figure 2E-F and were normalized to that of Ctl-1 cells in normoxia. For panels B-D, relative ion levels represent specific mass isotopomer fractions normalized to that of control cells in normoxia. [file 2049-3002-1-22-S4.jpeg]

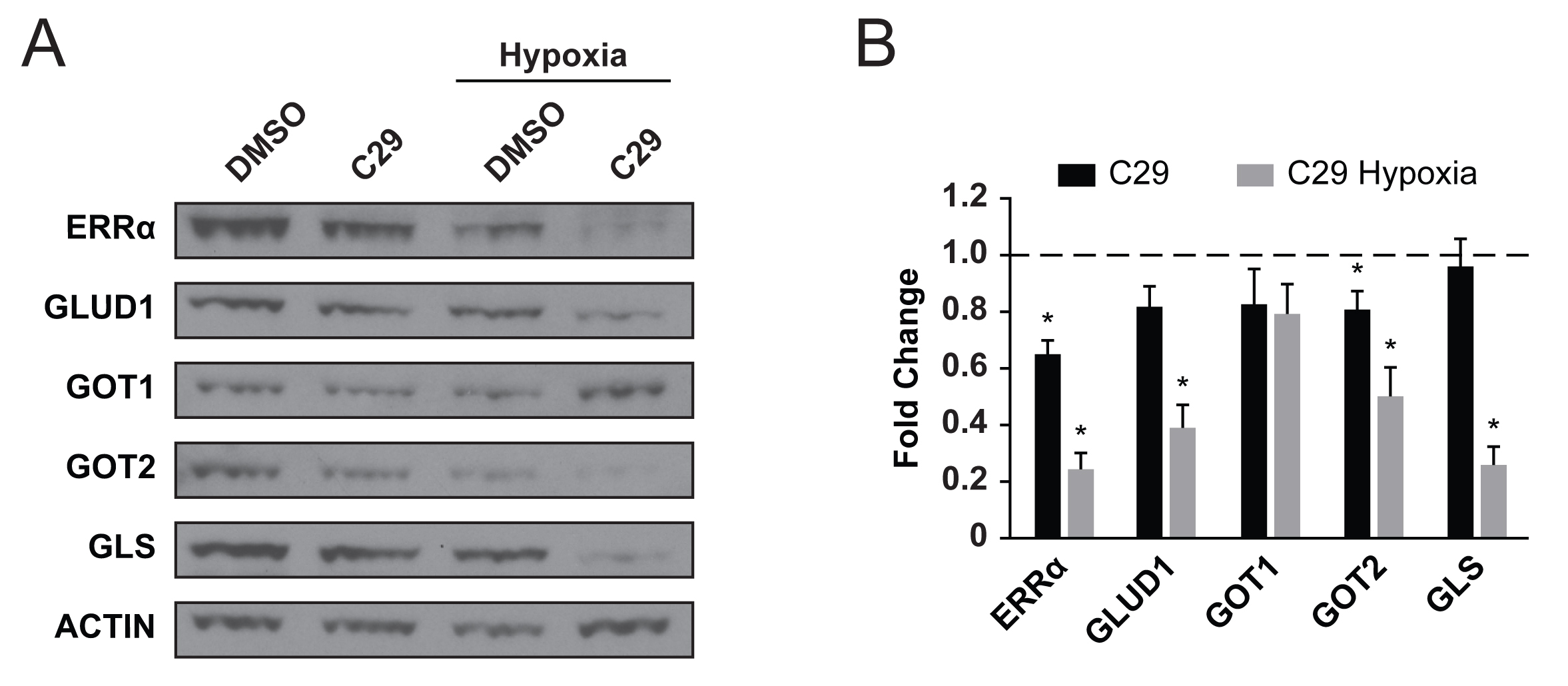

Supplement: Additional file 5: Figure S3 — ERRα modulates the expression of glutamine metabolism enzymes (A) Representative western blot for ERRα and select glutamine metabolism enzymes in SK-BR-3 cells treated with ERRα inhibitor C29 and cultured in either normoxia or hypoxia for 24 h (B) Quantification of five independent western blot experiments, normalized to ACTIN levels. Data are presented as means of C29-treated SK-BR-3 cells relative to control (DMSO) ± S.E.M., n = 5. *P <0.05, paired Student's t-test. [file 2049-3002-1-22-S5.jpeg]

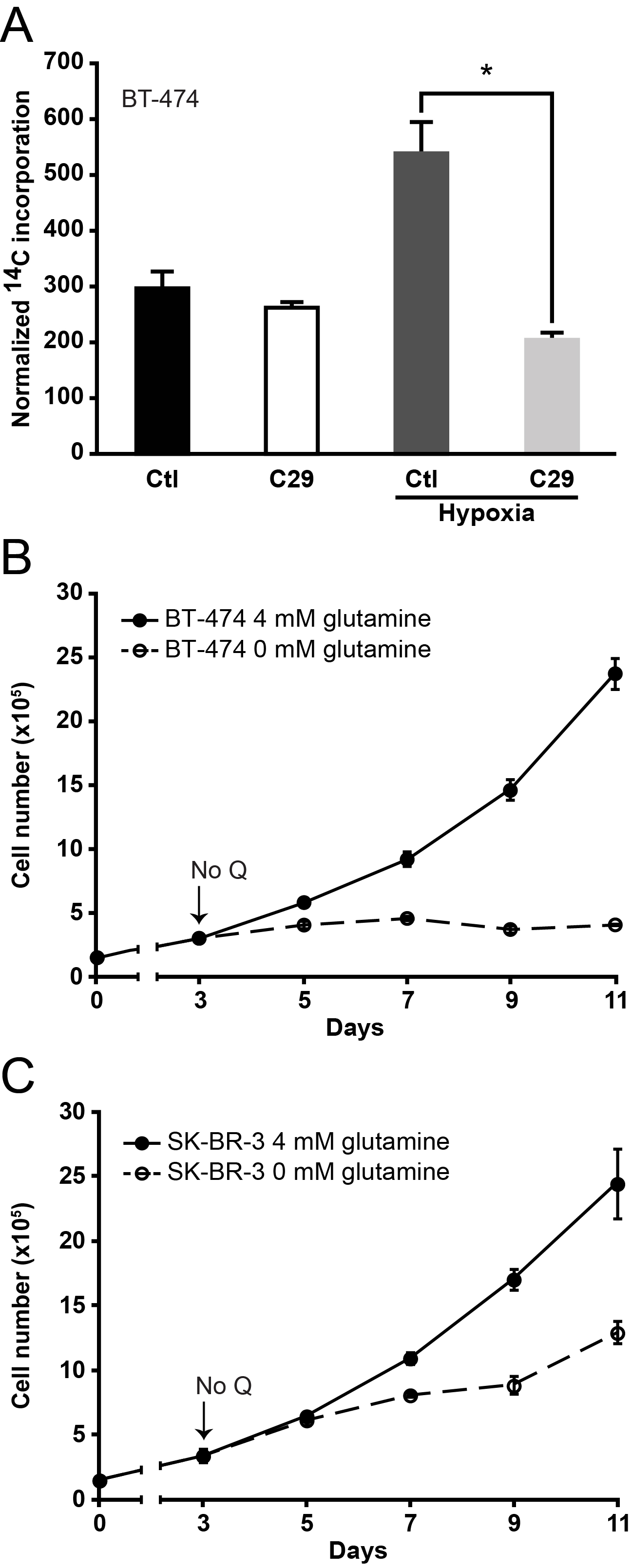

Supplement: Additional file 6: Figure S4 — ERRα promotes glutamine-mediated lipogenesis, and glutamine deprivation limits the proliferation of human ERBB2+ breast cancer cell lines. (A) Incorporation of 14C into lipids from trace [U-14C]-glutamine in BT-474 cells under normoxia or hypoxia treated with C29 or DMSO control. Counts were normalized for cell number and expressed relative to the count of control cells in normoxia. Data are presented as means ± S.E.M., n = 4. *P <0.05, paired Student's t-test. (B) Proliferation of BT-474 cells in the presence or absence of glutamine. Data are presented as means ± S.E.M., n = 3. (C) Proliferation of SK-BR-3 cells in the presence or absence of glutamine. Data are presented as means ± S.E.M., n = 3. [file 2049-3002-1-22-S6.jpeg]
